# Partial substitution of nitrate by chloride in fertigation recipes allows for lower nitrate input in hydroponic lettuce crops

**DOI:** 10.3389/fpls.2024.1411572

**Published:** 2024-07-23

**Authors:** Damianos Neocleous, Dimitrios Savvas, Evangelos Giannothanasis, Georgia Ntatsi

**Affiliations:** ^1^ Laboratory of Plant Nutrition, Department of Natural Resources and Environment, Agricultural Research Institute, Nicosia, Cyprus; ^2^ Laboratory of Vegetable Production, Department of Crop Science, Agricultural University of Athens, Athens, Greece

**Keywords:** hydroponics, nitrate, chloride, nutrient uptake, growth, chloride modelling

## Abstract

The management of nitrogen (N) fertilization is of fundamental importance in hydroponics. To reduce the supply of nitrate (NO_3_^-^) in fertigation recipes for Batavia lettuce crops grown in closed hydroponics, partial replacement of nitrate by chloride (NO_3_^-^/Cl^-^) at different ratios but with the same equivalent sum was experimentally tested. The experiment included four nutritional treatments in the replenishment nutrient solution, particularly T1; 0.7 mM Cl^-^/19 mM NO_3_^-^, T2; 2 mM Cl^-^/17.7 mM NO_3_^-^, T3; 4 mM Cl^-^/15.7 mM NO_3_^-^ and T4; 6 mM Cl^-^/13.7 mM NO_3_^-^. The results showed that reducing nitrate supply combined with equivalent increase in chloride application gradually reduced the gap between nitrate input and nitrogen uptake concentrations, with the smallest differences occurring in T4 treatment, which reduced the nitrate concentration in the drainage by 50%. The tested treatments led to very small variations in plant water uptake, production of fresh biomass and nutritional quality, which is justified by the proper functioning of key physiological mechanisms, such as stomatal conductance, which was followed by an increased efficiency of nitrogen use up to 25% (kg fresh biomass kg^-1^ N supply). The steady level of C/N ratio in the plant tissue irrespective of NO_3_^-^/Cl^-^ supply ratio points to sufficiency in photosynthetic products and adequacy in the supply of nitrogen, although leaf Cl^-^ content increased up to 19.6 mg g^-1^ dry weight in the lowest NO_3_^-^/Cl^-^ treatment. Nutrient uptake concentrations were determined as follows: 13.4 (N), 1.72 (P), 10.2 (K), 3.13 (Ca), 0.86 (Mg, mmol L^-1^), 27.8 (Fe), 5.63 (Mn), 5.45 (Zn) and 0.72 (Cu, μmol L^-1^). This study suggests that replacing 30% of NO_3_^-^ supply with Cl^-^ in fertigation recipes for hydroponic lettuce crops reduces leaf nitrate content without affecting physiological processes, growth, and quality, verifying in parallel the role of chloride as a beneficial macronutrient. Finally, a relationship between Cl^-^ uptake and its concentration in the root zone solution was established enabling the simulation of chloride to water consumption.

## Introduction

A large amount of nitrogen (N) is generally required in intensive production systems. Lettuce is a crop characterized by high N input recommendations in soilless culture reaching concentrations up to 19 mmol L^-1^, with nitrate-nitrogen (NO_3_-N) accounting for 85-90% of the total supply (Sonneveld and Voogt, 2009). Although this concentration may render high yields, high nitrate supply may result in high nitrate emissions to the environment and high nitrate concentrations in the edible parts which may breach either environmental (Nitrates Directive, 1991) or health-related restrictions (European Commission, 2011), respectively. In particular, accumulation of nitrate-nitrogen in fertigation effluents occurs when its supply exceeds plant needs and accumulation in plant tissue occurs when its uptake exceeds nitrate reduction and assimilation into organic-N, e.g. proteins and chlorophylls (Taiz and Zeiger, 2002). To overcome this problem in hydroponic lettuces and other leafy vegetable crops, nitrate lowering strategies have been adopted in several cases aiming mostly in limiting N availability in the nutrient solution before harvest (Conversa et al., 2021). In some other cases, environmental (e.g. radiation and temperature) or nutritional factors (e.g. biofertilizers) were suggested to enhance assimilation of nitrogen i.e., reduction and incorporation into organic-N (Gianquinto et al., 2013). In some other trials the antagonistic relationship between chloride (Cl^-^) and nitrate (NO_3_^-^) ions in the nutrient solution has been tested, aiming to reduce either nitrate uptake or vacuolar nitrate storage by plants (Carillo and Rouphael, 2022). However, all these strategies are not consisted in their efficiency due to contradictions in the growing conditions and other endogenous factors (e.g. species, harvest time, etc.). Therefore, growers are reluctant when it comes to their adaptation, as they are concerned about reduced crop yield. Therefore, to adopt an appropriate strategy, the role of an individual factor in the process has to be well understood. For example, the use of chloride in fertigation recipes in closed hydroponic systems serves also the objective of reduced N inputs in greenhouses, and is in line with the new multifunctional properties of chloride when separated from sodium (Raven, 2017; Colmenero-Flores et al., 2019).

N is a crucial element in both processes of photosynthesis i.e., light harvesting (component of chlorophylls) and CO_2_ fixation (component of Rubisco enzyme). On the other hand, photosynthetic carbon skeletons are used in nitrogen assimilation pathway. Therefore, photosynthetic- and N-metabolism are tightly bound and the enrolment of any individual factor in the process has to maintain an appropriate C/N balance in plants (Zheng, 2009). Vacuolar nitrate represents a significant part of total nitrogen in many plants (up to 30%), thus its reallocation for metabolic use instead of storage as an osmoticum is important for the regulation of nitrogen assimilation rates (Fan et al., 2009). In this context, Tischner (2000) reported vacuolar nitrate concentrations up to 100 mM, whereas 5 mM cytosolic concentrations were considered sufficient to retain the nitrate uptake rate in Chenopodiaceae plants. Thus, chloride transportation into the vacuole for osmoregulation purposes may restrict the need for nitrate deposition to vacuoles to maintain osmotic balance with the cytosol (He et al., 2017). In such a case, it is expected that physiological processes and crop yields are maintained even when the nitrogen supply is reduced, which finally enhance agronomic nitrogen use efficiency (i.e., lower nitrogen requirements to produce the same yield). Thus, chloride amendments in hydroponic nutritional schemes at appropriate levels (Wege et al., 2017) may exhibit synergistic rather than antagonistic interactions with nitrate supply favoring nitrogen utilization in lettuce plants as has been reported for some other plants (Franco-Navarro et al., 2016; Rosales et al., 2020; Neocleous et al., 2021). In commercial hydroponic crops, chloride is contained in sufficient quantities in irrigation water and agrochemical compounds (e.g. inorganic fertilizers) and thus, its addition to nutrient solutions is not needed (Sonneveld, 2002). Chloride has a micronutrient role in the photosynthetic function associated with water oxidation in PSII and regulation of enzyme activity. However, its concentrations within the plant tissues may rise up to levels similar with those of macronutrients (2-20 mg g^-1^ dry weight, Xu et al., 1999) when present at high concentrations in the external solution. At high external concentrations chloride is taken up freely by the roots (Sambo et al., 2019), and much of this is compartmentalized into the vacuole and cytosol (Papadopoulos and Hao, 2002). Thus, there is a notion that there are other functions of chloride within plants in addition to its role as a micronutrient in photosynthesis, which may be not essential but they are beneficial for plants. These include its role as an osmoticum in plant cells, as well as its role in the regulation of stomatal aperture, chloroplast performance and ionic charge balance (Franco-Navarro et al., 2016; Raven, 2017).

Considering this background, the present study aims to (i) study the synchronic application of nitrate (19, 17.7, 15.7, and 13.7 mM NO_3_^-^) and chloride (0.7, 2, 4 and 6 mM Cl^-^) compensated in an equivalent manner, thus not disturbing total ionic balance in the replenishment nutrient solution; (ii) identify possible beneficial effects of chloride on key physiological parameters in hydroponic lettuce crops enabling a better utilization of nitrogen in such systems; (iii) define new strategies of fertilization in hydroponic crops. To attain these objectives, firstly nutrient uptake concentrations (nutrient to water uptake ratio) were defined in all N/Cl treatments. Secondly, the effect of combined application of chloride and nitrate on physiological processes (e.g., stomatal conductance, mineral nutrition) was quantified, allowing for the complete recycling of the nutrient solution with no detrimental effects on yield (biomass production) and quality (antioxidant molecules, chlorophylls). Last but not least, chloride accumulation in relation to water consumption by lettuce plants in a closed hydroponic system was modelled. To do so, calibration and validation of existing empirical models was performed.

## Materials and methods

### Plant material - growing conditions

The experiment was conducted in a plastic-covered greenhouse at the Agricultural Research Institute of Cyprus (lat.: 34°45′N/long.: 33°20′E/elevat.: 40 m absl). Lettuce plants (*Lactuca sativa* var. Batavia) were transplanted to twenty independent closed-loop hydroponic NFT (Nutrient Film Technique) channels (length: 6 m, width: 0.25 m, height: 0.1 m) resulting in a planting density of 25 plants per sq. meter. Nutrient solution (NS) management was based on a programmed addition of nutrients instead of adding them based on the electrical conductivity of the recycled nutrient solution. The fertigation head thus produced a replenishment nutrient solution (RNS) based on the concept of expected mean nutrient uptake concentrations (UCs; mass of nutrient per volume of water absorbed) previously defined in the literature (e.g. Sonneveld and Voogt, 2009; Savvas and Neocleous, 2019). To do so, standard solutions of fertilizers were not added directly to the recirculated solution but through a fertigation head to the primary irrigation water to form the RNS and stored in a tank. From these tanks, the RNS was introduced directly to the recycled NS in each NFT circuit. The RNS was mixed with the recycled solution in a catchment tank placed at the end of each group of channels (experimental unit) to compensate for the water and nutrients absorbed by the plants. The injection of RNS was controlled through a floater adjusted to keep a constant volume of recirculating NS in the hydroponic circuits (experimental units). The pH in the recirculating NS was adjusted daily to 5.6-5.7. The NS in all NFT circuits was continuously recirculating day and night. Greenhouse inside temperature and humidity were maintained between 12-26°C and 55–75%, respectively, recording outside mean global solar radiation values from 11.2 (Autumn-Winter, AW) to 21.6 MJ m^-2^ d^-1^ (Spring, SP). Two lettuce crops were cultivated during Spring 2023 (SP crops, the second crop was a repetition of the first one) with the aim to study the effect of combined application of chlorides and nitrates on plants. The same experimental layout was used to grow two more crops during Autumn 2023 and Winter 2024 (AW crops) with the aim to simulate root zone chloride accumulation.

### Experimental treatments

Synchronic application of nitrate and chloride (NO_3_^-^/Cl^-^) ions at different ratios resulting in an equivalent total concentration, thus retaining the total ionic balance in the replenishment nutrient solution (RNS) were fitted in four nutritional treatments. T1; 0.7 mM Cl^-^/19 mM NO_3_^-^, T2; 2 mM Cl^-^/17.7 mM NO_3_^-^, T3; 4 mM Cl^-^/15.7 mM NO_3_^-^ and T4; 6 mM Cl^-^/13.7 mM NO_3_^-^. The concentrations of all other nutrients in the RNS were identical in all treatments (see **Table 1**). Thus, the EC in the RNS remained unchanged (2.63 dS m^-1^), irrespectively of the treatment. Chloride amendments in the irrigation water, which originally contained 0.7 mM Cl^-^, were attained by adding appropriate amounts of calcium chloride (CaCl_2_). Nutritional treatments were arranged in a randomized complete block design and replicated four times with two external rows in each side serving as guard plants. Each hydroponic circuit corresponded to one experimental unit. Experimental treatments commenced immediately after transplanting and lasted up to crop termination.

**Table 1:** Electrical conductivity (EC) and nutrient concentrations in the replenishment nutrient solution (RNS) supplied to lettuce crops (Batavia type), grown in a closed hydroponic system.

Parameter	Unit	Composition of the replenishment solution			
		T1	T2	T3	T4
EC	dS m^-1^	2.63	2.63	2.63	2.63
pH		5.6	5.6	5.6	5.6
K^+^	mmol L^-1^	11	11	11	11
Ca²^+^	mmol L^-1^	4.5	4.5	4.5	4.5
Mg²^+^	mmol L^-1^	1	1	1	1
NH_4_^+^	mmol L^-1^	1.25	1.25	1.25	1.25
SO_4_²^−^	mmol L^-1^	1.18	1.18	1.18	1.18
NO_3_^−^	mmol L^-1^	19	17.7	15.7	13.7
H_2_PO_4_^−^	mmol L^-1^	2	2	2	2
Fe	μmol L^-1^	40	40	40	40
Mn^++^	μmol L^-1^	5	5	5	5
Zn^++^	μmol L^-1^	4	4	4	4
Cu^++^	μmol L^-1^	0.75	0.75	0.75	0.75
B	μmol L^-1^	30	30	30	30
Mo	μmol L^-1^	0.7	0.7	0.7	0.7
Si	mmol L^-1^	0	0	0	0
Cl^−^	mmol L^-1^	0.7	2	4	6
Na^+^	mmol L^-1^	1.09	1.09	1.09	1.09
HCO_3_^−^	mmol L^-1^	0.25	0.25	0.25	0.25
K/(K+Ca+Mg)	mol/mol	0.67	0.67	0.67	0.67
Ca/(K+Ca+Mg)	mol/mol	0.27	0.27	0.27	0.27
Mg/(K+Ca+Mg)	mol/mol	0.06	0.06	0.06	0.06
N/K	mol/mol	1.84	1.72	1.54	1.36
NH_4_^+^-N/Total-N	mol/mol	0.06	0.07	0.07	0.08

The concentration of Cl^-^ in the primary water was 0.7, and the pH of the nutrient solution was set at 5.6 using nitric acid.

Nutrient solution compositions provided to hydroponic lettuces in the four nutritional strategies i.e., T1, T2, T3 and T4. T1 corresponds to standard nitrate concentration in the replenishment nutrient solution (RNS) and T2, T3 and T4 correspond to partial replacement of nitrate (NO_3_^-^) by chloride (Cl^-^) at different rates but in an equivalent manner in the RNS. Chloride was provided in the form of calcium chloride.

### Determination of nutrient uptake concentrations

The uptake concentrations (UCs) of selected macro- (N, P, K, Ca, Mg, mmol L^-1^) and micro-nutrients (Fe, Mn, Zn, Cu, μmol L^-1^), as well as of chloride (Cl; mmol L^-1^), were determined using two different estimation methods. The first method was based on the concentrations of nutrients measured in the recirculating nutrient solution at the beginning and the termination of each crop, which were used to estimate nutrient removal (disappearance) from the system due to plant uptake, in combination with the cumulative water consumption by plants. The obtained data were fitted in the following mass balance equation (Neocleous and Savvas, 2015; Neocleous and Savvas, 2016):


(1) $$UC_{x}=\frac{V_{s}(C_{xin}-C_{xf})+(V_{w}C_{xad})}{V_{w}}$$

where, *V_s_* is the volume of the recirculating nutrient solution in each hydroponic unit (L); *V_w_* is the cumulative water consumption of plants (L) for the studied period; *C_xin_* and *C_xf_* (mmol L^–1^) are the initial and final concentrations of the *x* nutrient in the recirculating nutrient solution, respectively; and *C_xad_* denotes the concentration of the *x* nutrient in the replenishment nutrient solution- (RNS, mmol L^–1^). The UC of total N depicts the sum of nitrate- and ammonium-nitrogen UC.

The second method was based on nutrient recovery from dry plant biomass which was divided by the water consumed by the plants to obtain the mass of nutrient per volume unit of water absorbed by the plants. According to this concept, the mean nutrient uptake concentrations were calculated using the following mathematical equation:


(2) $$UC_{x}=\frac{\left(C_{xl}B_{xl})+(C_{xr}B_{xr}\right)}{V_{w}}$$

where *C_xl_* and *C_xr_* denote the leaf and root nutrient concentration (mmol g^-1^ dry weight), respectively, *B_xl_* and *B_xr_* denote the leaf and root dry weight (g plant^-1^), respectively, and *V_w_* denotes the total water consumption by plants (L plant^-1^).

To feed Equations 1 and 2 with the required data, whole plants and samples of the nutrient solutions (150-200 ml) from each experimental unit were collected at the beginning and at the end of the crop. The daily water uptake was recorded regularly by the calibrated filling containers in order to calculate the consumption of nutrient solution in each experimental unit (water losses were negligible). At the same time, the electrical conductivity (EC), pH and temperature of the nutrient solution were measured using a Multimeter MM40+ (Crison Instruments, Spain) instrument. Representative leaf samples were oven-dried at 72°C to a constant weight and finely ground. The dry residue was proportioned to fresh biomass to determine dry matter content.

### Measurements

The concentration of nutrients in NS samples and plant tissues were determined based on a procedure that has been previously described by Neocleous et al. (2021) based on the methods summarized in Estefan et al. (2013). The nutrients were extracted from the leaf samples using a wet digestion procedure. The concentration of organic nitrogen in plant tissue was determined after Kjeldahl digestion (Mills and Jones, 1996) in a Kjeltec 2400 automatic analyzer. In nutrient solutions and aqueous extracts of plant tissues, Ca, Mg, Fe, Mn, Zn and Cu were determined using an atomic absorption spectrophotometer (Solaar M Series, Thermo Elemental, Cambridge, UK). Potassium was measured using a flame photometer (Model 420, Sherwood Scientific, Cambridge, UK) and P (vanadate-molybdate method) by a UV/VIS spectrophotometer (Lambda 35, Perkin Elmer; Waltham, MA, USA). The same spectrophotometer was used to determine the absorbances of NO_3_^–^ and NH_4_^+^–N ions in the nutrient solution samples. Chloride was extracted from leaf samples using hot water. The determination of Cl^-^ was done by silver nitrate titration method in the presence of potassium chromate. Lettuce yield (i.e., fresh leaves) was determined in each experimental unit at crop termination and nitrogen use efficiency (NUE) was defined as total leaf biomass per total nitrogen supply for each experimental unit (kg fresh biomass kg^-1^ N supply). Stomatal conductance measurements (AP4, Delta-T Devices, Cambridge, UK) were employed before harvesting in 6 plants per replication. Leaf carbon to leaf nitrogen ratio (C/N; mass of carbon per mass of nitrogen) was measured using elemental analyzer EA3000 (Euro Vector, Milan, Italy). Total sugar (glucose + fructose) and ascorbic acid content were measured using Merck (Darmstadt, Germany) assay kits and nitrate content in leaves was quantitatively determined by the salicylic acid method originally reported by Cataldo et al. (1975). Leaf chlorophyll content was measured in acetone extracts using equations from the literature (Lichtenthaler, 1987). The described methodology by Neocleous and Ntatsi (2018) was employed for estimating total phenolics, reducing potential (ferric reducing antioxidant power; FRAP assay) and radical scavenging activity (2,2-diphenyl-1-picrylhydrazyl; DPPH assay) in lettuce leaves.

### Modelling the accumulation of chloride in the root environment

To determine the relationship between chloride uptake by lettuce plants and its concentration in the drainage solution, standard mathematical relationships of linear i.e., *C_xu_=aC_xs_* or exponential type i.e., *C_xu_=aC_xs_^b^* (where *C_xu_* is the uptake Cl^-^/water ratio and *C_xs_* is the concentration of Cl^-^ in the root environment, see Sonneveld et al., 1999) were used to fit the experimental data. These equations were used as models to perform regression analysis using the pair of values from all treatments and replications. The estimated model parameters were then introduced into differential equations which were solved using the Runge–Kutta method to simulate chloride accumulation as a function of plant water consumption. The differential equations employed, and the methodology used to solve these, have been previously described in detail by Savvas et al. (2005, 2007). Briefly, the increase of chloride concentration in the closed system over the increase of cumulative water consumption relates to the difference between input (external concentrations) and output of chloride (plant uptake concentrations) over the volume of the recycled solution. Finally, to test the reliability of the prediction capacity of the model, the simulation data were evaluated against measured data from a lettuce crop grown in another experiment by using linear regression analysis.

### Statistical analysis

Preliminary analysis did not render any differences between the different crops in most of the cases. Therefore, data were pooled over crops, and analyzed by applying one way analysis of variance (ANOVA) using the statistical system SAS (ver. 9.2, SAS Institute Inc. Cary, NC, USA). When an ANOVA test was significant, means were compared using either Duncan’s Multiple-Range Test or Sidak’s T-test at a significance level *P ≤ 5%*, for data at calculation method level and between the results of the two methods, respectively. Data presented in graphs are means of four replications ± standard errors drawn using GraphPad Prism (ver. 5.0; GraphPad Software; San Diego California, USA). Regression analysis was performed to fit experimental data.

## Results

### Mineral nutrition, agronomical and physiological responses

The ratio between the mass of nutrient uptake and the volume of water consumed by the plants, commonly termed “uptake concentrations” (UCs), did not differ significantly between the two methods used for its estimation, for most nutrients (**Table 2**). However, the method based on the removal of P, Ca and Fe from the recirculating NS resulted in higher UCs compared to those obtained based on the total amounts of P, Ca and Fe that were recovered from the lettuce biomass. This can be explained by the fact that these ions may form precipitates or ion pairs that are not measurable, thereby rendering higher apparent UCs than the true ones. Nevertheless, apart from these three nutrients, both methods resulted in comparable values for all other nutrients studied (**Supplementary Figure 1**). Hence, the results can be safely adapted in reference nutrient solution compositions for hydroponic lettuce crops and as input data in fertigation tools (Neocleous and Savvas, 2022; Savvas et al., 2023). The validity of the results (UC values) at different environmental conditions is also supported by their good fitting to the 1:1 relationship (**Supplementary Figure 2**) between nutrient UCs determined at different seasons.

**Table 2:** Influence of the calculation method and the synchronic application of nitrate (NO_3_^-^) and chloride (Cl^-^) at different rates (T1, T2, T3, T4) in the replenishment nutrient solution (RNS) supplied to closed hydroponic lettuce crops (Batavia type) grown in two springtime experiments on nutrient uptake concentrations (mmol L^-1^).

Data pooled over experiments										
Treatments	N	P	K	Ca	Mg	Fe	Mn	Zn	Cu	Cl
**Method**										
Solution	13.4	1.72a	10.2	3.13a	0.86	27.8a	5.63	5.45	0.72	1.68
Biomass	12.7	1.18b	9.73	2.85b	0.83	24.6b	5.82	5.59	0.79	1.62
Significance	NS	***	NS	**	NS	***	NS	NS	NS	NS
Solution data pooled over experiments										
NO_3_^-^+Cl^-^ (mM)	N	P	K	Ca	Mg	Fe	Mn	Zn	Cu	Cl
19 + 0.7 (T1)	15.1a	1.71	10.3	3.17	0.89	28.4	5.65	5.43	0.74	0.51a
17.7 + 2 (T2)	13.6b	1.69	10.2	3.16	0.89	28.5	5.68	5.53	0.79	1.10b
15.7 + 4 (T3)	12.6c	1.74	10.2	3.04	0.82	27.3	5.72	5.55	0.79	1.98c
13.7 + 6 (T4)	12.3c	1.76	10.1	3.16	0.83	27.0	5.49	5.30	0.78	3.13d
Significance	***	NS	NS	NS	NS	NS	NS	NS	NS	***

T1 corresponds to standard nitrate concentration in the replenishment nutrient solution (RNS) and T2, T3 and T4 correspond to synchronic application of nitrate (NO_3_^-^) and chloride (Cl^-^) at different rates in the RNS as follows: T1; 0.7 mM Cl^-^/19 mM NO_3_^-^, T2; 2 mM Cl^-^/17.7 mM NO_3_^-^, T3; 4 mM Cl^-^/15.7 mM NO_3_^-^ and T4; 6 mM Cl^-^/13.7 mM NO_3_^-^. Chloride was provided in the form of calcium chloride. Values are means of four replicates. Significance of F: NS, not significant and * P< 0.05, ** P< 0.01, *** P< 0.001. When ANOVA was significant, means were compared using either Duncan’s Multiple-Range Test or Sidak’s T-test at a significance level P< 0.05, for data at calculation method level and between the results of the two methods, respectively. Different letters within a column indicate significant differences.

**Figure 1:**
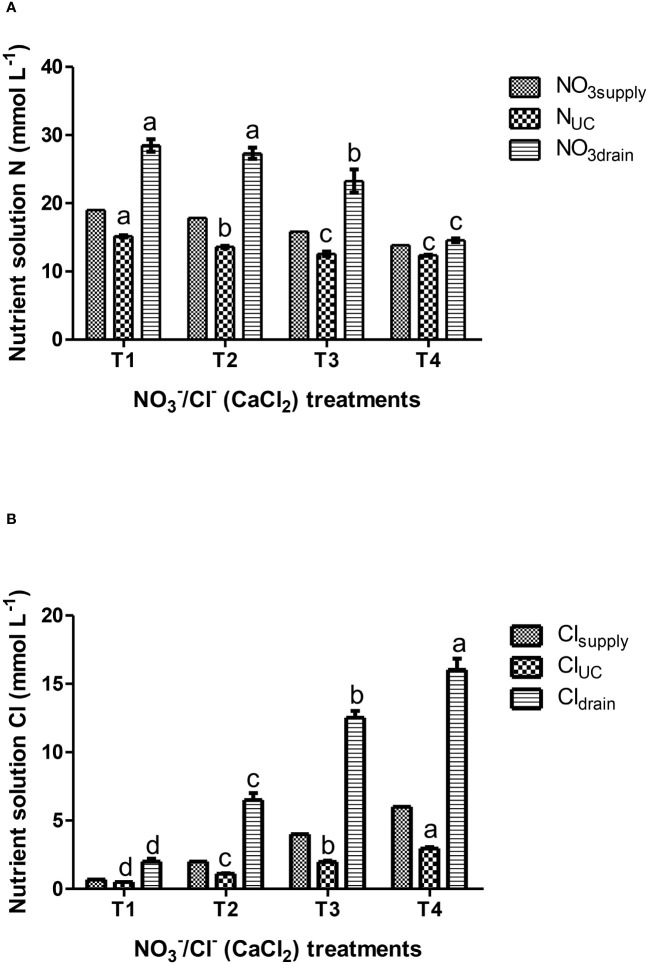
Effects of nutrient solution compositions provided in hydroponic lettuces by four nutritional treatments i.e., T1, T2, T3 and T4 characterized by synchronic application of nitrate and chloride at different rates (same equivalent sum) on: **(A)** solution N data (NO_3_ supply, N uptake concentration-UC, NO_3_ in the drainage); **(B)** solution Cl data (Cl^-^ supply, Cl^-^ uptake concentration-UC, Cl^-^ in the drainage). T1 corresponds to standard nitrate concentration in the replenishment nutrient solution (RNS) and T2, T3 and T4 correspond to synchronic application of nitrate (NO_3_^-^) and chloride (Cl^-^) at different rates in the RNS as follows: T1; 0.7 mM Cl^-^/19 mM NO_3_^-^, T2; 2 mM Cl^-^/17.7 mM NO_3_^-^, T3; 4 mM Cl^-^/15.7 mM NO_3_^-^ and T4; 6 mM Cl^-^/13.7 mM NO_3_^-^. Chloride was provided in the form of calcium chloride. Data presented in graphs are means of four replications ± standard errors. Means followed by different letters for the same column indicate significant differences according to Duncan’s multiple range test at a significance level *P*< 0.05.

To evaluate the impact of synchronic application of nitrate and chloride (NO_3_^-^/Cl^-^) on nutrient UCs, solution data were pooled over crops and reanalyzed as a single-factor experiment. The results in **Table 2** show significant differences in the UCs of nitrogen and chloride. The UCs of the other nutrients did not differ significantly between the different treatments. The results also showed that reducing nitrate supply under conditions of increased chloride ions in the nutrient solution gradually reduced the gap between nitrate input and nitrogen uptake concentrations by lettuce plants, with the smallest differences occurring in T4 treatment (6 mM Cl^-^/13.7 mM NO_3_^-^), which reduced nitrate concentrations up to 50% in the drainage solution (**Figure 1A**) compared to the control treatment T1 (0.7 mM Cl^-^/19 mM NO_3_^-^). Regarding plant water absorption and solution electrical conductivity (EC), it was observed that tested N/Cl treatments had no significant impact either on plant water uptake or on EC value in the root environment, which averaged 3.5 dS m^-1^ for the most of the growth period. However, at the end of the cropping cycle, EC rose to higher levels (avg. 4.5 dS m^-1^), with nitrate and chloride ions contributing the two-third of the total ionic strength (**Figures 1A, B**). This led to non-significant (*P<0.05*) differences in the production of fresh plant biomass among N/Cl treatments, which consequently was followed by an increased efficiency of nitrogen use (kg fresh biomass kg^-1^ N supply) by 25% in the lowest N/Cl treatment. Indeed, key physiological mechanisms of the plant, such as stomatal conductance (g_s_) were not influenced by the tested variations in NO_3_^-^ and Cl^-^ concentrations, recording values higher than 250 mmol H_2_O m^–2^ s^–1^ (**Table 3**). In good correspondence, no limitations in photosynthetic products (glucose and fructose) and a steady level of the C/N ratio were succeeded (**Table 3**). Furthermore, the leaf nitrate content decreased only when reducing the NO_3_^-^ concentration to 2/3 of the standard recommendations in the fresh nutrient solution, which corresponds to T4 treatment. By contrast, leaf organic N concentration which provides a reliable estimation of plant N status, remained unaffected by the applied treatments (N_leaf_, **Table 3**). Similarly to N, the leaf concentrations of other nutrients remained unaffected by the different nutritional treatments. The mean leaf macronutrient concentrations N, P, K, Ca and Mg amounted to 37.5, 8.1, 79.1, 13.2 and 2.9 mg g^-1^ dry weight, while those of micronutrients, particularly Fe, Mn, Zn and Cu amounted to 390, 117, 122 and 12 µg g^-1^ dry weight. Overall, the leaf nutrient status remained close to the optimal range suggested for this crop species (Gianquinto et al., 2013). On the other hand, lettuce plants treated with the highest Cl^-^ concentrations increased leaf Cl^-^ content following the Cl^-^ supply concentrations (**Table 2**), reaching levels similar to those of macronutrients (19.6 mg g^-1^ dry weight, **Table 3**). The quality effect of synchronic application of nitrate and chloride (NO_3_^-^/Cl^-^) ions on leaves was not significant and averages were as follow: dry matter (5.7%), sugar content (3.2°Brix), titratable acidity (0.1% citric acid), ascorbic acid (AA; 152 mg L^-1^), total phenolics (0.14 mg Gallic Acid g^-1^ fresh weight); FRAP values (0.25 μmol AA g^-1^ fresh weight); and DPPH (3.2 mg AA equiv. 100 g^-1^ fresh weight). Leaf chlorophyll was similar in the different treatments and averaged 4.26 µg ml^-1^ (3.04 µg Chla and 1.21 µg Chlb).

**Table 3:** Effects of nutrient solution compositions provided in hydroponic lettuces by four nutritional treatments i.e., T1, T2, T3 and T4 characterized by synchronic application of nitrate and chloride at different rates (same equivalent sum) on: fresh yield biomass (FY, g plant^-1^); total water uptake (WU, L plant^-1^); electrical conductivity in the drainage during plant growth (EC_grow_, dS m^-1^); electrical conductivity in the drainage at crop termination (EC_final_, dS m^-1^); stomatal conductance (g_s_, mmol H_2_O m^-2^ s^-1^); nitrogen use efficiency (NUE, kg FY kg^-1^ N supply); mass of carbon per mass of nitrogen (C/N ratio); glucose and fructose (Glu+Fru, mg g^-1^ fresh weight); leaf tissue nitrate content (LNC, mg NO_3_^-^ kg^-1^ fresh weight); leaf N- (N_leaf_, % dry weight); and leaf chloride-content (Cl_leaf_, % dry weight).

Data pooled over experiments											
NO_3_^-^+Cl^-^ (mM)	FY	WU	EC_grow_	EC_final_	g_s_	NUE	C/N	Glu+Fru	LNC	N_leaf_	Cl_leaf_
19 + 0.7 (T1)	404	5.1	3.5	4.1	300	300a	10.7	7.1	2135a	3.8	0.7a
17.7 + 2 (T2)	350	5.0	3.6	4.6	265	280a	10.9	7.1	2145a	3.8	0.9a
15.7 + 4 (T3)	389	5.0	3.7	4.6	273	353b	11.0	7.2	2113a	3.7	1.4b
13.7 + 6 (T4)	399	5.2	3.6	4.4	292	372b	11.1	7.3	1950b	3.8	2.0c
Significance	NS	NS	NS	NS	NS	***	NS	NS	*	NS	***

T1 corresponds to standard nitrate concentration in the replenishment nutrient solution and T2, T3 and T4 correspond to synchronic application of nitrate (NO_3_^-^) and chloride (Cl^-^) at different rates as follows: T1; 0.7 mM Cl^-^/19 mM NO_3_^-^, T2; 2 mM Cl^-^/17.7 mM NO_3_^-^, T3; 4 mM Cl^-^/15.7 mM NO_3_^-^ and T4; 6 mM Cl^-^/13.7 mM NO_3_^-^. Chloride was provided in the form of calcium chloride. Values are means of four replicates. Significance of F: NS, not significant and * P< 0.05, ** P< 0.01, *** P< 0.001. When ANOVA was significant, means were compared using Duncan’s Multiple-Range Test at a significance level P< 0.05. Different letters within a column indicate significant differences.

### Model calibration and performance

**Figure 2:**
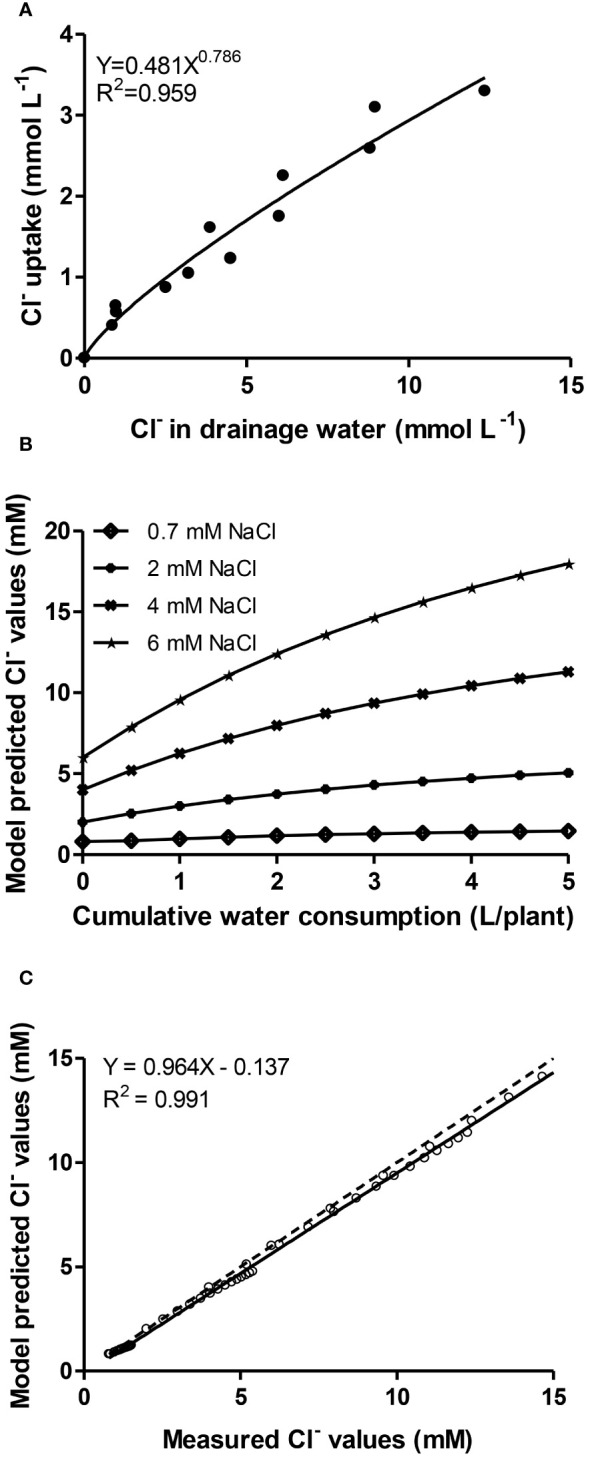
**(A)** Relationship (exponential of the type *Y=aX^b^* with coefficient of determination *R^2^ = 0.959*) between chloride uptake concentrations and its concentration in the drainage. Symbols depict pairs of values recorded in all chloride treatments with lettuce plants grown during the Autumn cropping period; **(B)** Model-predicted Cl^-^ concentrations in the root environment, in relation with cumulative water consumption of lettuce at 0.7-, 2-, 4- and 6-mM Cl^-^ in the irrigation solution; **(C)** Model-predicted values against measured data from a lettuce crop grown in another experiment (Winter cropping) performing linear regression analysis (solid line). The relation equation, regression coefficient and the 1:1 relationship between the two (dotted line) are presented.

**Figure 2A** shows the best fitted model (*Y= aX^b^*) according to the coefficient of determination (*R^2^ = 0.959*) between chloride uptake concentrations (i.e. the uptake ratio between the mass of Cl^-^ per volume of water absorbed by plants) and its concentration in the drainage. Symbols depict pairs of values recorded in all NO_3_^-^/Cl^-^ treatments during the Autumn cropping period. The estimation of model parameters (*a= 0.481, b= 0.789*) allows to use differential equations from the literature as mentioned elsewhere to retrieve model-predicted Cl^-^ concentrations in the root environment of lettuces, in relation with cumulative water consumption by plants at 0.7-, 2-, 4- and 6-mM Cl^-^ supply levels (**Figure 2B**). So, the model was calibrated for the whole cropping period of lettuce and a wide range of Cl^-^ external concentrations (0.7-17 mM) and cumulative water uptake up to 5 L plant^-1^ (**Figure 2B**). The prediction efficiency of the model was then validated against measured values from another experiment (Winter crop) providing, with all data in plot, a linear regression coefficient very close to unity (**Figure 2C**).

## Discussion

Nutrient uptake concentrations (UCs), defined as the mass of nutrient per volume of water absorbed, are based on two basic physiological functions of plants. The first is photosynthesis where the formation of carbon skeletons enables the assimilation of mineral nutrients to produce biomass. The second function is transpiration through the stomata of the leaves which is normally in equilibrium with the absorption of water by the roots. These two functions, although independent, are both controlled by the leaf stomata mechanism since carbon dioxide and water share the same transport pathway. Therefore, the ratio between them (nutrient to water uptake ratio-UCs) is characterized by relative stability over longer periods despite temporary fluctuations, since they are expected to be affected in a similar way. Indeed, in the present experiment, the UCs of most nutrients (except nitrates and chlorides, see below) were not significantly influenced by treatments (*P<0.05*) (**Table 2**). This can be explained by the fact that biomass, tissue nutrient concentrations (mentioned elsewhere) and water uptake, which are the factors defining UCs, were not affected (or they were similarly affected) by the nutritional treatments (**Table 3**). At any case, the plant nutritional status was within optimal ranges suggested in the literature (Gianquinto et al., 2013), which indicates that the increased Cl^-^ levels in the supplied NS (**Table 1**) did not restrict the nutrient availability. The UCs in the present study were determined by two independent methods and the results were similar for most nutrients apart from Ca, P, Fe (**Table 2**; **Supplementary Figure 1**). Furthermore, the UCs were similar under different environmental conditions (**Supplementary Figure 2**). Consequently, the UC estimated in the current study are considered credible and can be safely used as target values in reference nutrient solutions for recirculating systems or as standard input data for databases of decision support systems such as “NUTRISENSE” (Neocleous and Savvas, 2022; Savvas et al., 2023). Noteworthy, a balanced nutrient supply has been proven of primary importance for mitigating the effects of salinity in such environments (Neocleous et al., 2017).

The current results also showed that supplementing part of the nitrate supply with equivalent supply of chloride in lettuce grown in closed hydroponic systems gradually reduces the gap between nitrogen at the inlet (entering the system) and nitrogen at the output (uptake by the plants), with the smallest differences occurring in T4 treatment (6 mM Cl^-^/13.7 mM NO_3_^-^). Thus, this difference is related to the nitrate concentration in the drainage solutions, which recorded a 50% decrease in T4 compared to standard nitrate-nitrogen supply (T1, **Figure 1A**). Furthermore, the EC rose from 3.5 dS m^-1^ during the main cropping period to 4.5 dS m^-1^ at crop termination, however, the time exposure of the plants to the latter salinity level was probably too short to show any detrimental effects on growth (**Table 3**) or any visual symptoms. Previous studies have shown that a progressive exposure of plants to salinity due to a gradual accumulation of salts in the recycled solution is less detrimental compared to a rapid increase in the root zone solution (Bar-Yosef, 2008) as in the former case the plants can gradually adapt to the salinity conditions (Parida and Das, 2005). To ameliorate the negative effects of osmotic stress, it is well known that in salt treated plants the reduction of intercellular spaces to decrease transpiration rates can increase the density of mesophyll cells (Flexas and Medrano, 2002). In this case, the ratio of cell walls per unit area in the mesophyll tissue is increased, resulting in an increased mesophyll conductance to carbon dioxide (through cell walls), which in parallel can maintain water relations so as to sustain agronomical (e.g., fresh biomass) physiological (e.g., stomatal conductance) and biochemical (e.g., chlorophylls) mechanisms of adaptation in plants (Colmenero-Flores et al., 2019). Indeed, non-significant (*P<0.05*) variations in the production of fresh biomass and water uptake by lettuce plants between the tested treatments, can be justified by the proper functioning of key components of the physiological mechanisms of the plant, such as stomatal conductance (g_s_, **Table 3**) which were probably enhanced by the leaf morphological adaptations as mentioned above (not measured in the current study). In this context, stomatal conductance has been considered a reference parameter reflecting water imbalances in plants related with the control of xylem water and ion loading and salinity response functions (Flexas et al., 2004; Orsini et al., 2013), which is consistent with current results (**Table 3**). Particularly, current values of g_s_ (>250 mmol H_2_O m^-2^ s^-1^) were much higher to threshold’s values (100-150 mol H_2_O m^-2^ s^-1^, irrespectively of the maximum g_s_ attained by a given species), reported to be capable to sustain Rubisco activity (Flexas et al., 2004). Regarding chloride, increasing its supply levels in the RNS increases chloride concentrations in the root environment correspondingly, which entails increased uptake (**Figure 1B**) and accumulation of Cl^-^ ions at high concentrations in plant tissues (Cl_leaf_, **Table 3**). Particularly, lettuce plants supplied with T4 treatment (6 mM Cl^-^) increased leaf Cl^-^ content to macronutrient levels (19.6 mg g^-1^ dry weight) without detrimental effects. These concentrations were much higher than typical concentrations (0.1-0.2 mg g^-1^ dry weight; Wege et al., 2017), which would theoretically be toxic for non-tolerant plants to chloride (5-15 mg g^-1^ dry weight). However, the current results agree with the findings of Xu et al. (1999), who reported normal growth and appearance of lettuce plants with chloride concentrations in their leaves ranging from 2.8 to 19.8 mg g^-1^ dry weight, and concluded that prolonged chloride treatment in the low milli-molar range may result in high leaf accumulation with positive growth responses in lettuce and no stress symptoms (Colmenero-Flores et al., 2019). Thus, these results allow us to suggest that chloride, accumulating at high leaf concentrations under a prolonged chloride treatment (≤ 6 mM) without the presence of sodium at similar concentrations in the root zone, may have beneficial functions in lettuce Batavia plants. These include the use of Cl^-^ as an osmoticum within plant cells and its involvement in the maintenance of the ionic charge balance, the regulation of stomatal aperture, and chloroplast performance according to the literature (Franco-Navarro et al., 2016; Raven, 2017).

On the other hand, the reduced nitrate supply through partial substitution by chloride in the supplied NS to the tested levels (**Table 1**) was an effective strategy for reduction of the nitrate uptake (**Table 2**) and leaf nitrate content without reducing the organic-N content in plant tissues (**Table 3**). This indicates that (i) nitrogen assimilation processes resulting in biosynthesis of amino-acids and other reduced-N forms were not affected by the lower amount of nitrogen delivered and eventually absorbed (**Table 2**); (ii) the activity of nitrate assimilating enzymes present in the cytosol or chloroplasts was not restricted due to the occurrence of chloride ions (Liu et al., 2014). The mechanism explaining this response it may be related to a low level of cytoplasmatic Cl^-^ (Carillo and Rouphael, 2022) stimulated by a higher cytoplasmic extrusion of Cl^-^ in the vacuole through active processes (Colmenero-Flores et al., 2019). Consequently, replacing part of NO_3_^-^ with Cl^-^ in nutrient solutions at certain levels, reduces merely the uptake of NO_3_^-^ destined to function as osmoticum in vacuoles due to substitution of NO_3_^-^ by Cl^-^, while having no impact on the uptake of NO_3_^-^ reduced into organic N. The findings of the current study are in line with the beneficial role attributed to Cl^-^ ions per se in experiments with other plants (Colmenero-Flores et al., 2019; Neocleous and Savvas, 2022), indicating that Cl^-^ is a beneficial macroelement in Batavia lettuce crops, where it may act as an osmotic agent and preserves water relationships, increases nitrogen use efficiency and maintains physiological (e.g., stomatal conductance) and agronomical functions (biomass production, **Table 3**). Furthermore, the stable level of C/N ratio suggests sufficiency in photosynthetic products (glucose and fructose) and adequacy of nitrogen in plant metabolism to sustain optimal growth (**Table 3**). Indeed, the synthesis of nitrogen biomolecules (e.g., chlorophyll synthesis) and the proper function of metabolic pathways that contribute to nutritional quality (e.g., antioxidant molecules, see results session, Beauvoit et al., 2018) points towards adequacy in precursors for biosynthesis and carbohydrate availability under such conditions. In parallel, no increase in the antioxidant activity (reducing potential and radical scavenging activity) was observed, in relation with the increased concentration of chlorides (in the solution and within plants), which implies that when chloride is applied at concentrations up to 6 mM and its supply is not accompanied by sodium at similar concentrations, it should not be considered as an abiotic stress factor causing oxidative stress and free radical production in plants (Carillo and Rouphael, 2022). This is supported by the fact that the C- and N-assimilation pathway and the performance in photosynthetic products (glucose and fructose) do not reveal any limitations or possible failures in the electron transport chain. Overall, limiting nitrogen supply by one third of the standard recommendations (Sonneveld and Voogt, 2009) while substituting this with chloride did not affect the basic metabolic pathways and products supporting the primary and secondary metabolism in hydroponic lettuces. In consequence, the partial substitution of NO_3_^-^ by Cl^-^ in T4 treatment (lowest N/Cl) increased the efficiency of nitrogen use (kg fresh biomass kg^-1^ N supply) by 25% without detrimental effects on lettuce yield (**Table 3**).

Finally, the relationship between Cl^-^ uptake and its concentration in the root zone solution was described by the exponential equation *C_xu_=aC_xs_^b^* (where *C_xu_* is the uptake ratio Cl^-^/water and *C_xs_* is the concentration of Cl^-^ in the root environment). Appropriate calibration of the model parameters (*a= 0.481, b= 0.789*) entails a very good agreement (linear relationship with slope and regression coefficient close to unity) between simulated (model-predicted) values and measured values from another experiment. Although many trials have introduced the counteracting effect between chloride and nitrate in the nutrient solution (Carillo and Rouphael, 2022), no model relating chloride accumulation with the water consumption has been developed for lettuce up to date to our best knowledge. Thus, these results may be used to quantify the extend of nitrate substitution by chloride in nutrient solutions supplied to lettuce in closed hydroponic systems with the aim of reducing N input in intensive production systems.

Consequently, our results allow us to suggest partial replacement of NO_3_^-^ supply with Cl^-^ by one third in the fertigation recipes for hydroponic Batavia lettuce crops. Thus, establishing an optimal ratio in the replenishment nutrient solution reduces nitrogen inefficiencies allowing for complete recycling of the nutrient solution under raised Cl^-^ (solution, ≤6 mM) and tissue, ≤19.6 mg g^-1^ dry weight) concentrations retaining physiological processes, growth and quality, verifying the role of chloride as a beneficial macronutrient in Batavia lettuces. Consequently, developing smart cultivation techniques in soilless culture will eventually contribute to the reduction of water and nutrient inefficiencies in Mediterranean greenhouses.

## Conclusion

The replacement of 30% of the NO_3_^-^ supply with Cl^-^ in the fertigation recipes for closed hydroponic lettuces entails that part of nitrate fertilizer input can be replaced by chloride fertilizers (e.g. in the form of calcium chloride) without any specific negative effects on yield and quality (without deleterious effects on plants). In parallel, the partial substitution of NO_3_^-^ by Cl^-^ in closed hydroponic lettuce crops enhances N utilization by the plants and reduces NO_3_^-^ concentrations in the edible part and in the drainage. It seems that similar osmoregulation properties of nitrate and chloride enables reduction in vacuolar nitrate accumulation and its remobilization to retain total N assimilated in the plants. This reduces nitrogen inefficiencies without affecting primary and secondary metabolism, thus agronomical, physiological and biochemical responses in lettuce crops. The chloride model developed in this work ensures an efficient tool to be implemented to predict chloride accumulation in relation with water consumption. Finally, determining UCs for a wide range of environmental conditions serves also as a tool to establish hydroponic nutritional schemes for closed lettuce crops in Mediterranean greenhouses or can be embedded in decision support systems to readjust nutrient injection rates.

## Data availability statement

The original contributions presented in the study are included in the article/**Supplementary Material**. Further inquiries can be directed to the corresponding author.

## Author contributions

DN: Conceptualization, Data curation, Formal analysis, Funding acquisition, Investigation, Methodology, Project administration, Resources, Software, Supervision, Validation, Visualization, Writing – original draft, Writing – review & editing. DS: Conceptualization, Methodology, Project administration, Supervision, Validation, Visualization, Writing – review & editing. EG: Data curation, Investigation, Methodology, Writing – original draft. GN: Data curation, Investigation, Methodology, Writing – original draft.

## Supplementary material

Supplementary Figure 1 Impact of the calculation method (solution vs. biomass data) on the uptake concentrations (UCs) of selected nutrients with all data in plot. Symbols depict pair of values for each nutrient. Relation equation and regression coefficient are presented.

Supplementary Figure 2 Seasonal impact on the uptake concentrations (UCs) of selected nutrients with all data in plot. Linear regression analysis was performed between two cropping seasons (Spring vs. Autumn-Winter data). Symbols depict pair of values for each nutrient. Relation equation and regression coefficient are presented.
