# Supplementary figures and images for: Partial substitution of nitrate by chloride in fertigation recipes allows for lower nitrate input in hydroponic lettuce crops
Source: Front Plant Sci. 2024 Jul 23;15:1411572. doi: 10.3389/fpls.2024.1411572 (PMC11304451; doi:10.3389/fpls.2024.1411572)

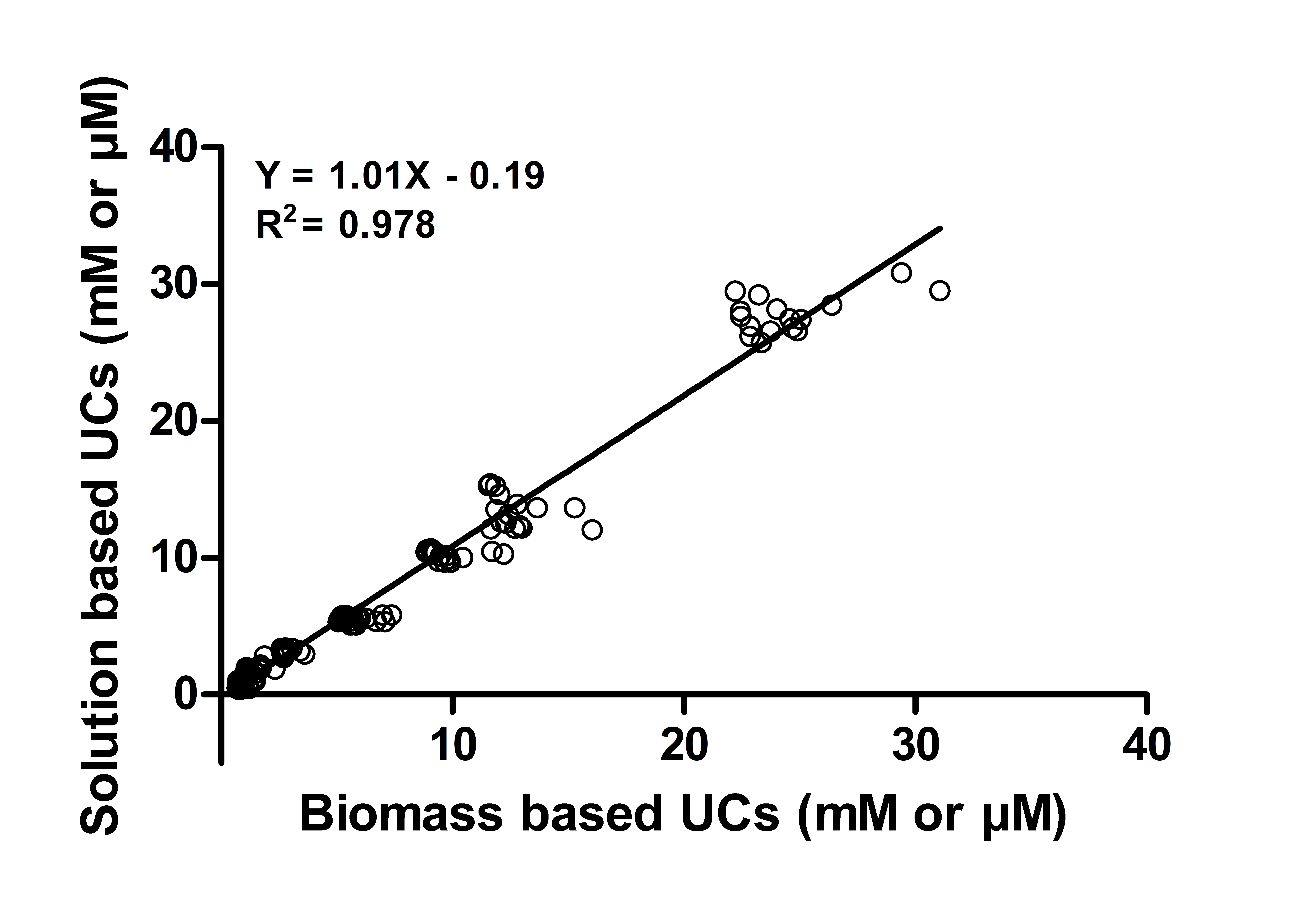

Supplement: Supplementary Figure 1 — Impact of the calculation method (solution vs. biomass data) on the uptake concentrations (UCs) of selected nutrients with all data in plot. Symbols depict pair of values for each nutrient. Relation equation and regression coefficient are presented. [file Image_1.jpg]

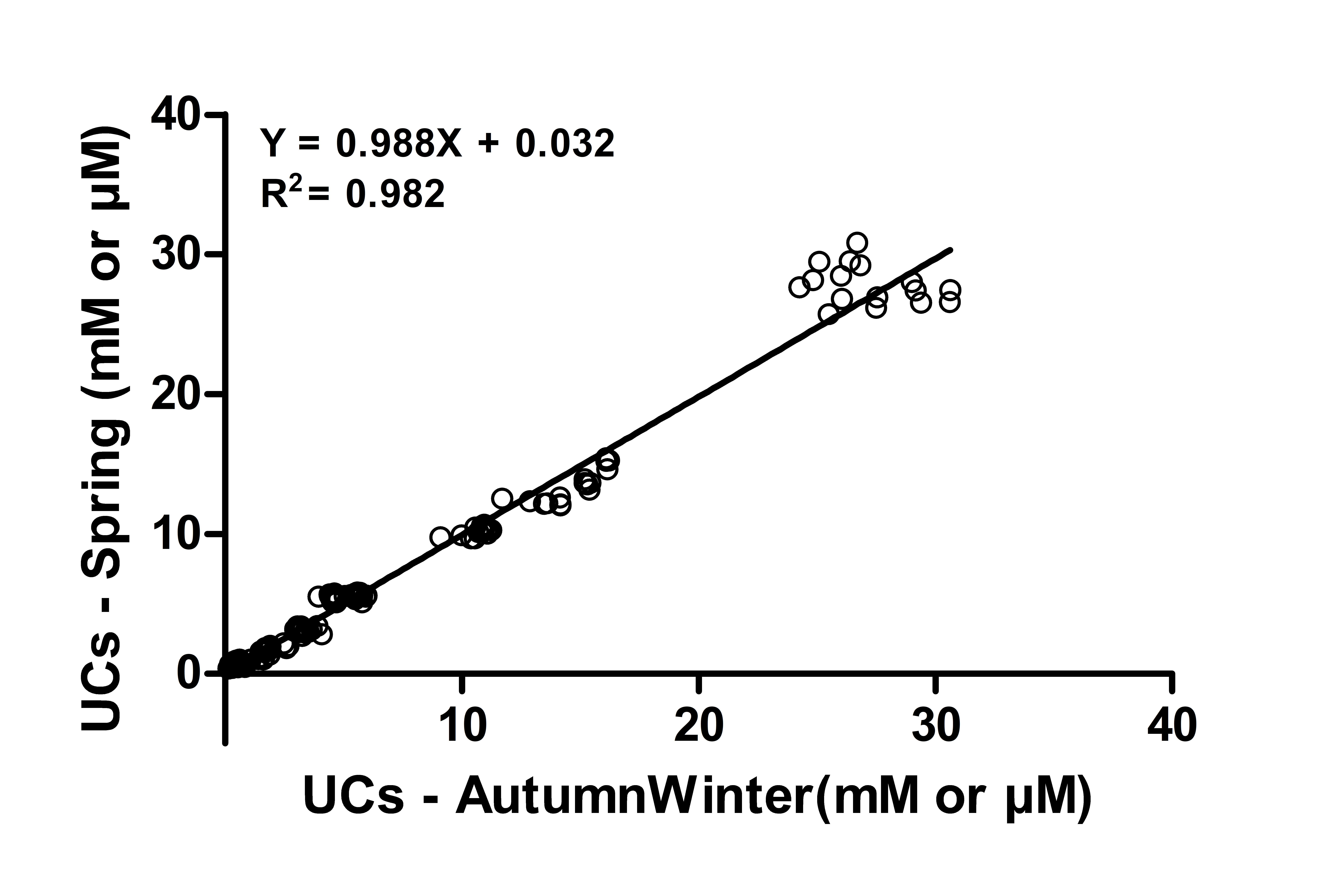

Supplement: Supplementary Figure 2 — Seasonal impact on the uptake concentrations (UCs) of selected nutrients with all data in plot. Linear regression analysis was performed between two cropping seasons (Spring vs. Autumn-Winter data). Symbols depict pair of values for each nutrient. Relation equation and regression coefficient are presented. [file Image_2.jpg]
